# Difficulties in using simulation to assess abdominal palpation skills

**DOI:** 10.1186/s12909-023-04861-6

**Published:** 2023-11-23

**Authors:** Xiaowei Xu, Haoyu Wang, Jingfang Luo, Changhua Zhang, Lars Konge, Lina Tang

**Affiliations:** 1 The Seventh Affiliated Hospital, Sun Yat-Sen University, Shenzhen, China; 2 Guangdong Academy for Medical Simulation (GAMS), Guangzhou, China; 3 School of Medicine, Sun Yat-Sen University, Shenzhen, China; 4 Copenhagen Academy for Medical Education and Simulation, Copenhagen, Denmark

**Keywords:** Abdominal palpation, Simulation, Assessment, Validity

## Abstract

**Objectives:**

Abdominal palpation is an essential examination to diagnose various digestive system diseases. This study aimed to develop an objective and standardized test based on abdominal palpation simulators, and establish a credible pass/fail standard of basic competency.

**Methods:**

Two tests were designed using the newly developed Jucheng abdominal palpation simulator (test 1) and the AbSim simulator (test 2), respectively. Validity evidence for both tests was gathered according to Messick’s contemporary framework by using experts to define test content and then administering the tests in a highly standardized way to participants of different experience. Different simulator setups modified by the built-in software were selected from hepatomegaly, splenomegaly, positive McBurney’s sign plus rebound tenderness, gallbladder tenderness (Murphy’s sign), pancreas tenderness, and a normal setup without pathologies, with six sets used in test 1 and five sets used in test 2. Different novices and experienced were included in the tests, and test 1 was also administered to an intermediate group. Scores and test time were collected and analyzed statistically.

**Results:**

The internal consistency reliability of test 1 and test 2 showed low Cronbach’s alphas of 0.35 and -0.41, respectively. Cronbach’s alpha for palpation time across cases were 0.65 for test 1 and 0.76 for test 2. There was no statistical difference in total time spent and total scores among the three groups in test 1 (*P*-values (ANOVA) were 0.53 and 0.35 respectively), nor between novices and experienced groups in test 2 (*P*-values (t-test) were 0.13 and 1.0 respectively). It was not relevant to try to establish pass/fail standards due to the low reliability and lack of discriminatory ability of the tests.

**Conclusions:**

It was not possible to measure abdominal palpation skills in a valid way using either of the two standardized, simulation-based tests in our study. Assessment of the patient’s abdomen using palpation is a challenging clinical skill that is difficult to simulate as it highly relies on tactile sensations and adequate responsiveness from the patients.

## Introduction

Abdominal palpation is an essential body examination to diagnose various digestive system diseases, e.g. liver cirrhosis, ascites, etc. [1]. However, most pre-interns and interns find it challenging to perform abdominal palpation accurately and effectively because it requires sophisticated manual skills and the ability to interpret a large volume of clinical information [1–3]. Therefore, it is essential to determine the optimal way to train abdominal palpation to ensure that students meet the requirements of clinical practice.

There are two essential processes in learning: practice and testing. Traditionally, body examination has been taught using the apprenticeship model where novices practice directly on patients supervised by a senior colleague [4]. Experienced faculty is necessary for supervising and providing feedback. However, ethical considerations, lack of supervisors, and increased concerns for patient safety make the practice opportunities shrink. Students can practice manual skills on each other or use standardized patients, but the lack of pathologies and resemblance to the real clinical scenario reduce the learning effect [5, 6]. Simulation-based training on physical phantoms and virtual-reality simulators would allow trainees to practice repeatedly in a standardized and completely safe environment until basic competency is acquired [6, 7]. Various abdominal simulators have been manufactured and used in the training of medical students, ranging from manikins with physical organs inserted into the cavity [8, 9] to models (ACDET’s ABSIM system) with computerized automatic control [6]. Practicing on the simulators have improved students’ abdominal examination skills on the simulators but it is unknown if skills are transferable to real patients.

The other crucial element in the learning process – testing of skills—also deserves our attention. Mastery Learning is a very efficient and recommended training method where trainees continue to practice until they have reached a pre-defined mastery level [10]. A good test with solid evidence of validity is a prerequisite for mastery learning [11]. The current standard of exploring a test requires validity evidence from five sources: content, response process, internal structure, relations with other variables, and consequences [12]. Assessments based on objective metrics provided by virtual-reality simulators have been used for other procedures to provide automatic, unbiased test results [13, 14], but to our knowledge, this has not been done for abdominal palpation. Hamm et al. used the AbSim simulator to do abdominal palpation competence assessment before and after training for 3rd year medical students, and they found that guided abdominal simulator practice increased medical students’ capacity to perform an abdominal examination. However, this study did not provide sufficient validity evidence for the simulation-based test that was used [6].

Abdominal palpation is complex and involves different depths of palpation and different organ-based maneuver skills. Appropriate pressure and speed are essential for effective abdominal palpation [15]. Force feedback has been increasingly used in simulation studies, which includes different modalities: visual feedback, auditory feedback, tactile feedback, and their combinations [16]. Hsieh et al. designed digital abdominal palpation devices to assess the pressure of palpation on healthy participants [15]. S. Riek et al. developed a haptic device to assess the haptic feedback forces in a simulator aiming to train the skills of gastroenterology assistants in abdominal palpation during colonoscopy [17]. Both these studies are enlightening and have paid attention to force feedback in the use of the simulator as a training model, but none of them have developed a valid abdominal palpation test using the simulators.

This study aimed to develop two objective and standardized tests based on two different abdominal palpation simulators, gather valid evidence for the tests, and establish credible pass/fail standards that can ensure basic competency before continuing to clinical practice.

## Methods

The first test was based on a newly developed simulator from our own center and the second test used a commercially available simulator. Validity evidence for both two tests was gathered according to Messick’s contemporary framework by employing experts to define tests (validity evidence for content) and then administering the tests in a highly standardized way (validity evidence for response process) to participants of different experience (validity evidence for internal structure, relationship to other variables, and consequences). To avoid a learning-by-testing effect, we used different participants for data collection for each test. The process was slightly different for the two tests due to differences in simulator content and practical experience gathered during data collection for the first test.

### The tests

**Fig. 1:**
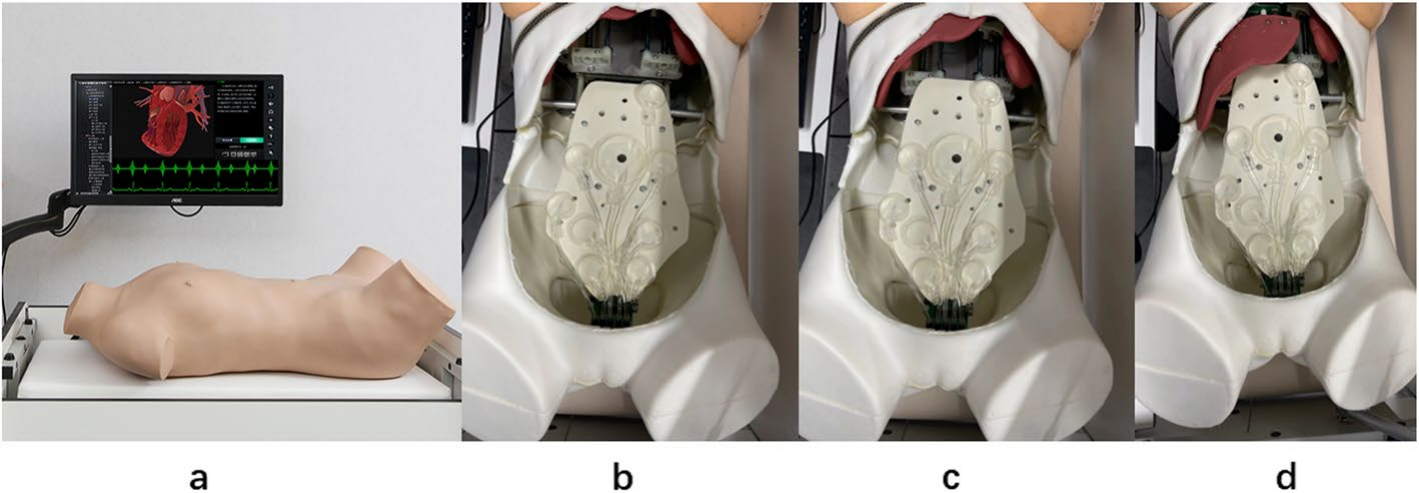
**a** External view of Jucheng simulator and its operation station. **b** Internal view during inhaling. **c** Internal view during exhalation. **d** Internal view when simulating hepatomegaly

**Fig. 2:**
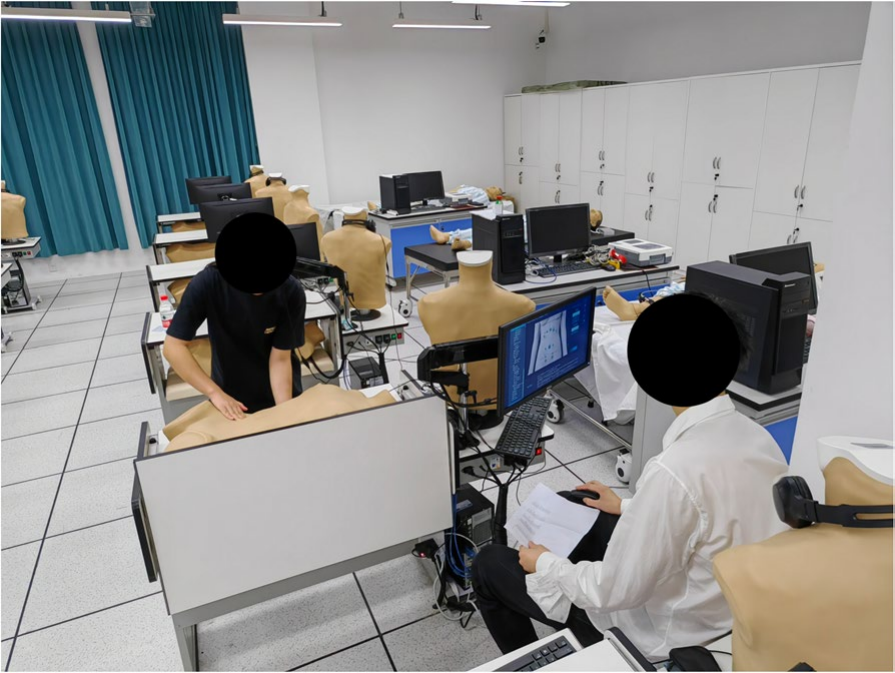
A student using the Jucheng simulator

The newly developed simulator (From Jucheng, Inc., Yingkou, China) used for the first test consists of a phantom with software that can change the size of the liver, gallbladder, and spleen, and the degree of tenderness and rebound pain of 14 tenderness points across the abdomen (Figs. 1 and 2).

The Jucheng simulator is a combination of a physical phantom and computer technology to allow training and assessment of abdominal examination skills. The phantom is an adult female half model, 76 cm*37 cm *22 cm, with TPE (thermoplastic elastomer) material on the skin, which can simulate different abdominal pathological signs accompanied with deep or shallow abdominal breathing. Hepatomegaly or splenomegaly can be simulated as the liver module or spleen module moves up and down, and the built-in pressure sensors can also be used to simulate tenderness and rebound pain in 16 different points. The phantom emits responsive vocalizations of pain, which can be relayed through either a built-in speaker or headphone. Figure 1a, b, c, and d show the external and internal views of the Jucheng simulator in various modes such as inhaling, exhalation and simulating hepatomegaly. Figure 2 shows a student using the Jucheng simulator.

**Fig. 3:**
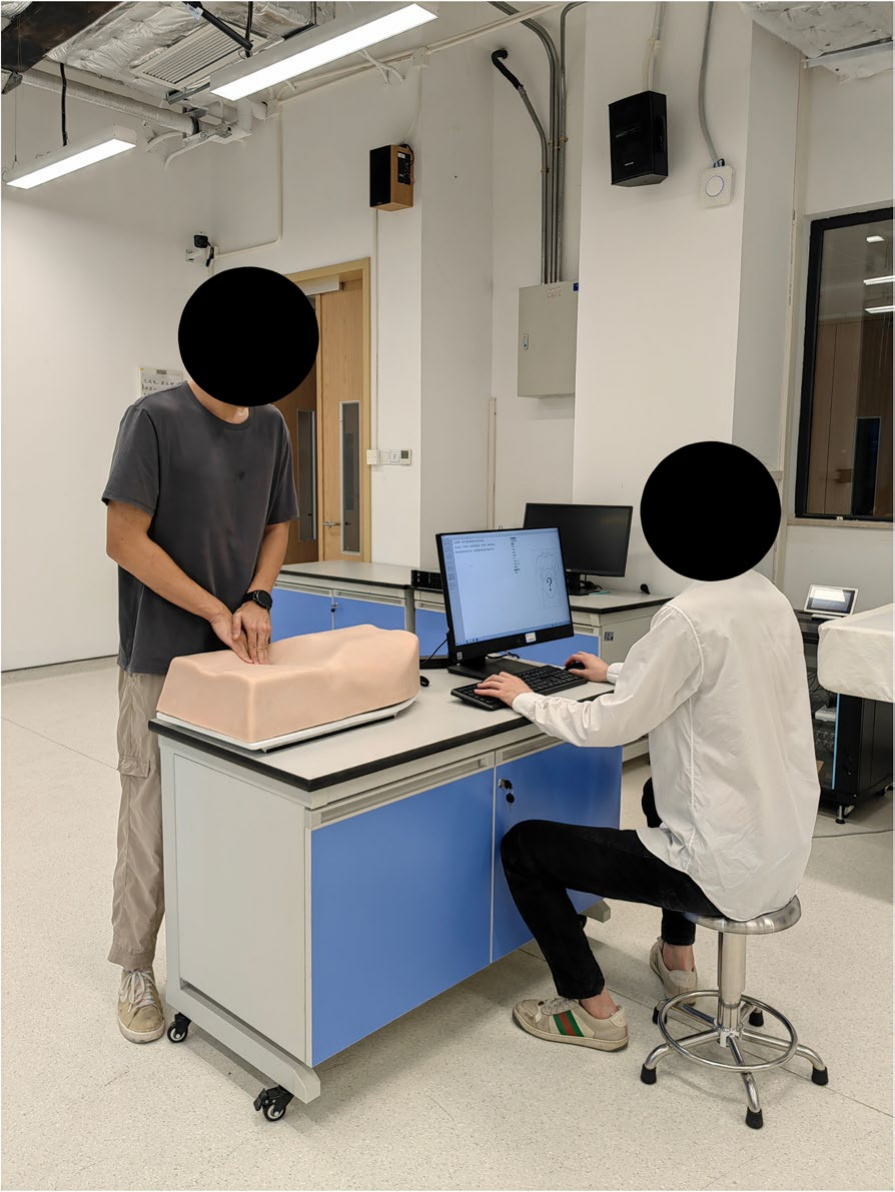
A student using the AbSim simulator

The second test was based on the AbSim abdominal simulator (ACDET, Inc., Fort Worth, Texas) that allows setting of the size of liver and spleen as well as the tenderness degree for different points across the abdomen (Appendix, Colon, Gallbladder, left Ovary, right Ovary, Pancreas, Urinary Bladder) (Fig. 3).

The AbSim simulator is a human physical examination training system composed of computer system and half of the physical examination abdomen model. It is 56 cm long, 35 cm wide, and 27 cm tall with silicon skin. The system can register the users' palpation force on the abdominal model with blue, gray, and red shown on the monitor.

In contrast to the AbSim simulator, the newly developed Jucheng simulator simulates breathing during the abdominal palpation, which could be beneficial for practicing hepatomegaly or splenomegaly detection. Besides, the Jucheng simulator exhibits superior volumetric dimensions and provides a heightened degree of versatility in representing a spectrum of hepatomegaly and splenomegaly severities, ranging from mild to severe. However, the skin of the Jucheng simulator has a harder feel than the AbSim simulator and it lacks real time force feedback on the monitor.

Experts in abdominal palpation, student education, and simulation-based assessment defined the content of the tests. The first test consisted of six different simulator setups (i.e. cases) modified by the built-in software: hepatomegaly, positive McBurney’s sign plus rebound tenderness, severe splenomegaly, positive Murphy’s sign, pancreas tenderness, and a normal setup without pathologies. The second test consisted of five different simulator setups including hepatomegaly, splenomegaly, gallbladder tenderness (Murphy’s sign), Appendix tenderness (McBurney’s sign) with rebound tenderness, and normal setup without pathologies.

#### The participants

Volunteering participants with different experiences in abdominal palpation were recruited. The novices were 12 fifth-year medical students from Sun Yat-sen University with no previous practical experience and the experienced group consisted of 12 physicians with more than three years of experience with abdominal palpation. The first test was also administered to an intermediate group consisting of 12 residents from the Departments of Internal Medicine, Surgery, Oncology, and Neurology at the Seventh Affiliated Hospital of Sun Yat-sen University. They had performed ≥ 10 abdominal palpations and had a maximum of one year of experience in abdominal palpation. Therefore, 36 participants (12 in each of the three groups) were included in data collection for test 1 and 24 participants (12 in each of the two groups) were included for test 2.

#### The testing process

Each participant came to the simulation center and signed informed consent. The order of the different cases in the tests (six and five cases, respectively) was randomized on a test sheet to avoid participants memorizing the cases and passing these on to the subsequent participants. A simulator assistant set up the simulator according to the test sheet and asked the participant to palpate the simulated abdomen and tick one box with the correct finding. Each case in the first test had ten different answer opportunities: hepatomegaly, discrete splenomegaly, severe splenomegaly, gastric tenderness, diffuse intense tenderness and rebound pain, appendix tenderness (McBurney’s sign) without rebound tenderness, appendix tenderness with diffuse rebound pain, appendix tenderness with distinct rebound tenderness, gallbladder tenderness (Murphy’s sign), gallbladder and gastric tenderness, and normal abdomen without pathologies. The cases in the second test had 12 different answer options: hepatomegaly, splenomegaly, gastric tenderness, appendix tenderness (McBurney’s sign) without rebound tenderness, appendix tenderness with rebound tenderness, gallbladder tenderness (Murphy’s sign), urinary bladder tenderness, colon left lower tenderness, ovary left tenderness, ovary right tenderness, pancreas tenderness, and normal setup without pathologies.

The simulator assistant did not offer any feedback or guidance to the participants during the tests.

#### Scoring

Each correct answer was awarded a score of one point resulting in possible maximum scores of six points in the first test and five points in the second test. The scoring was totally objective, i.e. based solely on simulator settings and the participants’ single-best-answer without room for interpretation. The time spent on palpating each of the six and five cases in the two tests was registered by the simulator assistant without pre-warning before and during the tests lest the participant feel rushed and stressed.

### Statistical analysis

An item analysis with item difficulty index and item discrimination index was done for the six cases in test 1 and for the five cases in test 2. The overall Cronbach’s alpha across items (i.e. internal consistency reliability) was calculated for both tests.

The total test scores and the total test time of the three groups in test 1 were compared using one-way analysis of variance (ANOVA) and the two groups in test 2 were compared using an independent samples T-test. One-way ANOVA and T-test were also used to compare the groups’ scores for each of the 11 cases in the tests.

All analyses were performed using the software package IBM Statistical Package for the Social Sciences (SPSS) version 25. *P*-values below 0.05 were considered statistically significant.

## Results

There was no statistical difference between male and female in each test an both of the two tests had age different among or between their groups (see Table 1). All participants completed the test without missing data (ensured by the simulator assistant).

**Table 1:** The demographic features of each group in test 1 and test2

Tests	Groups	Gender M/F	Chi-Square Tests	Age (Mean ± SD)	Age (Median)	*P*-Values
**Test 1**	**Novice**	**8/4**	**0.06**	**22.7 ± 0.9**	**23.0**	***P* < 0.001**
	**Intermediate**	**4/8**		**30.8 ± 2.8**	**30.5**	
	**Experienced**	**9/3**		**38.1 ± 7.2**	**39.0**	
**Test 2**	**Novice**	**6/6**	**0.34**	**22.0 ± 0.4**	**22.0**	***P* < 0.001**
	**Experienced**	**4/8**		**36.9 ± 4.5**	**36.0**	

Validity evidence for content was established by the developers of the two simulators and the experts in our group that picked relevant simulator settings to include as cases in each of the two tests. Validity evidence for the response process was ensured by the standardized administration of the test where the cases were administered in a randomized order by a simulator assistant that did not offer any feedback or guidance but only objectively noted down the time spent and the single-best answer from the list of answer opportunities.

The internal structure of total scores in test 1 showed a low Cronbach’s alpha of 0.35. Cronbach’s alpha for test 2 was negative (-0.41) due to a negative average covariance among items which violates the reliability model assumptions. Cronbach’s alphas for palpation time across cases were better, 0.65 for test 1 and 0.76 for test 2. The completion times for each test and group was listed in Table 2. No statistical difference of completion time was found in test 1, neither was test 2 except items of Gallbladder tenderness or Appendix tenderness in which experienced doctors were much faster than novice students.

**Table 2:** The *completion times* of the 11 cases in the two tests

	**Novices** **Completion time(s)**	**Intermediates** **Completion time(s)**	**Experienced** **Completion time(s)**	***P*-Values**
**Test 1 (Jucheng simulator used)**				
Hepatomegaly	**50.4 ± 30.1**	**49.3 ± 19.7**	**36.2 ± 12.7**	**0.23**
Positive McBurney’s sign + rebound tenderness	**44.3 ± 30.8**	**32.1 ± 16.4**	**33.8 ± 11.0**	**0.32**
Splenomegaly	**60.8 ± 52.2**	**75.0 ± 38.1**	**51.8 ± 30.5**	**0.39**
Positive Murphy’s sign	**62.5 ± 53.4**	**80.1 ± 55.7**	**83.6 ± 48.8**	**0.58**
Pancreas tenderness	**48.7 ± 24.7**	**46.3 ± 39.9**	**45.3 ± 25.6**	**0.96**
Normal abdomen	**56.4 ± 34.8**	**94.5 ± 44.5**	**72.2 ± 43.6**	**0.09**
**Test 2 (AbSim simulator used)**				
Hepatomegaly	**85.1 ± 34.4**	**-**	**72.6 ± 52.8**	**0.49**
Splenomegaly	**58.1 ± 27.9**	**-**	**71.1 ± 34.7**	**0.32**
Gallbladder tenderness	**73.3 ± 28.8**	**-**	**45.7 ± 33.5**	**0.04**
Appendix tenderness	**86.1 ± 40.2**	**-**	**48.1 ± 20.7**	**0.008**
Normal abdomen	**88.8 ± 48.1**	**-**	**69.8 ± 32.8**	**0.27**

The item difficulty indices and item discrimination indices of the 11 cases in the two tests are listed in Table 3.

**Table 3:** The item difficulty indices and item discrimination indices of the 11 cases in the two tests

	**Novices % correct**	**Intermediates % correct**	**Experienced % correct**	**Overall % correct**	**Disc. Index**	***P*-value**
**Test 1 (Jucheng simulator used)**						
• Hepatomegaly	58	92	67	72	0.21	0.18
• Positive McBurney’s sign + rebound tenderness	50	92	67	69	0.11	0.09
• Splenomegaly	58	58	50	56	0.14	0.90
• Positive Murphy’s sign	8	8	17	11	0.03	0.77
• Pancreas tenderness	17	0	8	08	0.08	0.36
• Normal abdomen	50	67	67	61	0.38	0.65
**Test 2 (AbSim simulator used)**						
• Hepatomegaly	58		67	63	0.23	0.69
• Splenomegaly	83		42	63	-0.04	0.04*
• Gallbladder tenderness	83		67	75	-0.32	0.37
• Appendix tenderness	67		92	79	-0.01	0.15
• Normal abdomen	67		92	79	-0.43	0.15

The relationship to other variables showed that neither test 1 nor test 2 were able to discriminate between groups. The novices, intermediates, and experienced spent 323 s (SD 159), 377 s (SD 155), and 323 s (SD 72), respectively, to perform test 1, and scored 2.42 points (SD 1.31), 3.17 points (SD 1.03), and 2.75 points (SD 1.36). *P*-values were 0.53 for total time spent and 0.35 for total score. For test two, the novices spent 391 s (SD 122) and the experienced spent 307 s (SD 138), *p* = 0.13. The novices scored 3.6 points (SD 1.1) and the experienced also scored 3.6 points (SD 0.67), *p* = 1.0. Table 1 shows the percentage of correct answers for each of the groups in each of the 11 cases. Only the splenomegaly cases on the AbSim simulator had a significant *p*-value (*p* = 0.04), but unfortunately this was because more novices (83%) than experienced (42%) identified this pathology.

Due to lack of discriminatory ability (and the low reliability), it was pointless to explore the validity evidence regarding consequences for the tests.

## Discussion

In this study, we failed to develop a valid abdominal palpation test to establish a credible pass/fail standard based on the newly developed Jucheng abdominal simulator and the AbSim abdominal simulator. Consistent negative results despite the use of two different phantom-based models and good consistency between the included content of the two independent tests, demonstrate the difficulty of developing a valid abdominal palpation test based on a phantom simulator. Phantom simulators might be suitable as training tools with educational value, but currently it is not possible to plan mastery learning training programs where everybody continue practicing until a pre-defined pass/fail level is met – simply because the simulator cannot be trusted to ensure clinical proficiency.

Studies like this with negative results are rare due to publication bias. However, it is not the first study about simulators failing to assess competency in a valid way. In 2017, Mills et al. reported that there was no correlation between attending surgeons’ simulator performance and expert ratings of intraoperative videos based on the Global Evaluative Assessment of Robotic Skills scale [18]. Although novice surgeons may put considerable effort into training on robotic simulators, performance on a simulator may not correlate with attending robotic surgical performance [18].

When it comes to a phantom-based abdominal palpation simulator, the reason for its failure as a test tool is not clear yet, but simulating this examination heavily relies on tactile sensations and adequate reactions from the “simulated patient” regarding tenderness, pain etc. [15]. This could have a part to play in the negative result of this study. Compared to abdominal palpation simulators equipped with tactile feedback [15–17], simulators used in surgery or endoscopy assessment have been widely used [19]. Valid tests based on simulation offer novice doctors an opportunity to practice the skill set necessary to perform laparoscopy exam or surgery efficiently, and could be used as an educational tool [20]. In these scenarios, tactile feedback is not so highly demanding as in the abdominal palpation simulators. Judging from the big gap between popular valid tests on the simulation-based laparoscopy and the relatively few studies of abdominal palpation simulation, we have reason to consider heavy reliance on tactile sensations as one of the reasons to explain our negative results.

Besides, we ought to pay attention to the “artificial effect” in the simulator “skin texture” which may also play a part in the failure of abdominal palpation as a test tool. Even though both the Jucheng and the AbSim simulators are equipped with silicon skin, their softness and elasticity are still not able to be compared with patient’s abdominal skin. But abdominal palpation highly relies on the delicate feeling of hands on touch of the “skin” of patients or simulators. Too hard or too soft both decreases the accuracy of palpation. Similar to abdominal palpation, the existing literature reveals a deficiency in the development of training methods for medical students in the examination of skin [6, 21]. The latest advances of skin simulation models have incorporated smart phone-based skin simulation models as a training tool, but the absence of substantial validity evidence underscores the challenges associated with simulations involving skin [22].

Medical simulators could be classified into compiler-driven types and event-driven types (standardized patients/care actors, hybrid simulation, and computer-based simulators) [23]. Per fidelity, medical simulators could be low-fidelity, medium-fidelity, and high-fidelity simulators [24, 25]. Both of the simulators used in this study are computer-based and virtual-simulated, with medium fidelity. Even with the objective limitation that simulators are unsuitable for ensuring abdominal palpation skills, they could still have value for training purposes. However, simulation training should be based on solid evidence of efficacy and proof of transfer of skills to real patients should be demanded [26].

However, this doesn’t mean the attempt to develop an abdominal palpation simulator suitable for testing should be stopped for good. Compared with simulated patients, virtual reality simulation of abdominal palpation offers more convenience for medical students and novice residents to practice, free of ethics issues. In the long run, virtual reality simulation benefits the trainees, the multidisciplinary team, and the hospital as a whole [23]. Immersive haptic force feedback technique could be considered and developed more, as well as the artificial intelligence assistance in mimicking real patients’ reactions to palpation [27].

## Conclusion

In conclusion, it was not possible to measure abdominal palpation skills in a valid way using either of the two standardized, simulation-based tests in our study. Assessment of the patient’s abdomen using palpation is a challenging clinical skill that is difficult to simulate as it relies highly on tactile sensations and adequate responsiveness from the patients. Additionally, this study underscores the urgent need and provides insights into the future direction for developing abdominal palpation simulators.

## Data Availability

The datasets used and/or analyzed during the current study available from the corresponding author on reasonable request.
